# Evolution of bioluminescence in Anthozoa with emphasis on Octocorallia

**DOI:** 10.1098/rspb.2023.2626

**Published:** 2024-04-24

**Authors:** Danielle M. DeLeo, Manabu Bessho-Uehara, Steven H.D. Haddock, Catherine S. McFadden, Andrea M. Quattrini

**Affiliations:** ^1^ Department of Invertebrate Zoology, National Museum of Natural History, Smithsonian Institution, Washington, DC, USA; ^2^ Department of Biological Sciences, Institute of Environment, Florida International University, Miami, FL, USA; ^3^ Institute for Advanced Research, Nagoya University, Nagoya, Japan; ^4^ Graduate School of Science, Nagoya University, Nagoya, Japan; ^5^ Monterey Bay Aquarium Research Institute, Moss Landing, CA, USA; ^6^ Dept of Ecology and Evolutionary Biology, University of California, Santa Cruz, CA, USA; ^7^ Department of Biology, Harvey Mudd College, Claremont, CA, USA

**Keywords:** coral, cnidarian, phylogenetics, genomics, phylogeny, deep sea

## Abstract

Bioluminescence is a widespread phenomenon that has evolved multiple times across the tree of life, converging among diverse fauna and habitat types. The ubiquity of bioluminescence, particularly in marine environments where it is commonly used for communication and defense, highlights the adaptive value of this trait, though the evolutionary origins and timing of emergence remain elusive for a majority of luminous organisms. Anthozoan cnidarians are a diverse group of animals with numerous bioluminescent species found throughout the world's oceans, from shallow waters to the light-limited deep sea where bioluminescence is particularly prominent. This study documents the presence of bioluminescent Anthozoa across depth and explores the diversity and evolutionary origins of bioluminescence among Octocorallia—a major anthozoan group of marine luminous organisms. Using a phylogenomic approach and ancestral state reconstruction, we provide evidence for a single origin of bioluminescence in Octocorallia and infer the age of occurrence to around the Cambrian era, approximately 540 Ma—setting a new record for the earliest timing of emergence of bioluminescence in the marine environment. Our results further suggest this trait was largely maintained in descendants of a deep-water ancestor and bioluminescent capabilities may have facilitated anthozoan diversification in the deep sea.

## 1. Introduction

Bioluminescence is a fascinating phenomenon in which visible light is emitted from living organisms. It has been documented in over 16 phyla and 900 genera of metazoans with the majority of observations occurring in marine environments [1,2], where it is commonly used to locate or lure food, attract mates and/or evade predation [3,4], contributing to an organism's overall fitness. The ubiquity of bioluminescence in marine environments highlights its importance to ocean life, while the evolutionary convergence of this trait across the tree of life is indicative of its fitness value [3]. The adaptive value of bioluminescence can be further quantified and understood by studying the evolution of this trait through deep time. Examining the emergence of bioluminescence and species diversification associated with bioluminescent capabilities allows for a better understanding of the origin and evolution of this phenomenon throughout the geological history of life on earth.

Bioluminescence is defined as the chemical reaction between a substrate (luciferin) and an enzyme (luciferase) resulting in light emission in a living organism. Luciferins and luciferases are identical and homologous among lineages that share a common evolutionary origin of bioluminescence, respectively, with exceptions in some marine organisms. A recent study suggested that the ability to bioluminesce has evolved more than 100 times independently (i.e. greater than 100 origins) across the tree of life [1,3–6]. However, detailed natural history focused on the evolution of bioluminescence is limited to just a few groups of animals where robust phylogenies are available (e.g. beetles, ostracods and fish). One of the earliest-known origins of bioluminescence in the marine environment occurred around 267 million years ago (Ma) in ostracods [7]. For ray-finned fishes, which exhibit widespread and diverse modes (e.g. symbiotic, endogenous and kleptoproteinic) of bioluminescence, bioluminescence is predicted to have evolved at least 27 times, with origins between the Mesozoic (150–65 Ma) and Cenozoic (65 Ma to present day), across species-rich deep-sea, meso- and bathypelagic lineages (i.e. dragonfishes and lanternfishes) [8]. The evolutionary origins and timings for the appearance of bioluminescence among the majority of the luminous species remain uncertain or scarcely studied.

Cnidarians in the class Anthozoa are morphologically and genetically diverse and among the oldest clades of animals [9,10]. This group is composed of ecologically important foundation species that are found throughout the oceans [11]. Various morphological trait innovations have been linked to the ecological success of Anthozoa throughout the Phanerozoic Eon (approx. 541 Ma to present), including colonial growth forms, precipitation of crystalline aragonite or calcite skeletons and establishment of symbioses with photosynthetic dinoflagellates [11]. The evolution of additional complex phenotypic traits among Anthozoa, including bioluminescence, remains enigmatic due to limited information and access to a diverse taxonomic range of specimens, though recent efforts have attempted to address these questions using experimental and high-throughput sequencing approaches [12]. Though the role of bioluminescence in Anthozoa remains unclear, it has been hypothesized to be used for communication with commensal and/or obligate associates [13], prey attraction and predator avoidance [4]. Therefore, the innovation of this trait may have contributed to the diversification and subsequent ecological success of various anthozoan lineages, particularly in the light-limited deep sea (depths greater than 200 m) because there are perceivably fewer luminous taxa in coastal and shallow areas (e.g. [14]).

Several groups of anthozoans have been documented as bioluminescent, but little is known about the ecological roles, molecular mechanisms and evolutionary origins of bioluminescence. Within the Anthozoa, octocorals (soft corals, gorgonians, sea pens etc.) are among the major groups of luminous animals found on the seafloor [15], with the first report of bioluminescence described in *Parasphaerasclera (=Eleutherobia) grayi* (Thomson and Dean, 1931) from the Solomon Islands [16]. Prior to this study, 33 luminous genera were reported from Octocorallia while only six had been reported from Hexacorallia [12]. Both coelenterazine, a luciferin found from the sea pansy *Renilla* (Octocorallia) and coelenterazine-dependent luciferase activity were found from luminous anthozoans of both Octocorallia and Hexacorallia classes (e.g. [12]). The occurrence of coelenterazine, however, does not necessarily indicate a common luminous ancestor of Anthozoa, since this luciferin can be obtained through the food web and is widely used among jellies (i.e. scyphozoans, hydrozoans and ctenophores), fishes, echinoderms and crustaceans [4,17,18]. On the other hand, luciferase-coding genes are inherited, and sequence similarity can provide evidence of a single origin of bioluminescence. The *Renilla-*type luciferase was detected by immunoblotting and genomic investigations in various octocorals but not in hexacorals, though a luciferase-like gene was detected via transcriptome-mining of a cerianthid (Hexacorallia) [12]. These results suggest that bioluminescent traits, with potentially different genetic bases for light emission, may have evolved independently among the sub-classes Octocorallia and Hexacorallia.

Exploring the diversity of bioluminescence in Octocorallia in a phylogenetic context can provide insight into factors potentially influencing the gain and/or loss of this trait. Here, we summarize the prevalence of bioluminescence among Anthozoa and use a phylogenomic approach to explore the evolution of this trait among the species-rich and phenotypically diverse class Octocorallia (approx. 3500 species), which encompasses a majority of the known bioluminescent anthozoans to date. As octocorals appear to share identical and homologous bioluminescent substrates and enzymes, we hypothesized that a single origin of bioluminescence likely exists for this group. Due to the high prevalence of bioluminescence in deep-sea habitats, we further hypothesized that the evolution of bioluminescence may be correlated with a deep-sea (greater than 200 m) lifestyle. Thus, for bioluminescent taxa inhabiting shallow depths, we hypothesize that bioluminescence evolved before those lineages colonized and subsequently diversified in shallow waters. Our study provides evidence for a single origin of bioluminescence in Octocorallia and infers the age of the phenomenon across these ancient animals, setting a new record for the earliest timing of the emergence of bioluminescence in the marine environment.

## 2. Methods

### (a) Bioluminescence across Anthozoa

The recently published fossil-calibrated phylogeny [10] (1729 loci from 234 anthozoans, consisting of representatives from all orders and most families) was used to map bioluminescent traits and depth categories across Anthozoa. Bioluminescence was opportunistically tested by agitating specimens with forceps in a dark room and the bioluminescence was detected by the naked eye or recorded by digital camera or camera phone (electronic supplementary material, figure S1). The specimens were collected by dredging off the coast of Sugashima, Japan in 2023 or by Remotely Operated Vehicle during the R/V Celtic Explorer expeditions in 2014 and 2016 and the R/V Ron Brown expedition in 2019. While false negatives for bioluminescence were possible given the stress associated with sampling, the authors only tested corals that appeared to be in good condition. Many species that were not bioluminescent were repeatedly tested across multiple field seasons. A literature search was performed to confirm additional records, or lack thereof, of bioluminescence within each representative genus. Taxa were categorized based on a scale of 0–1 (0: *Non-bioluminescent or unlikely*; species or closely related species tested with negative results; 1: *Likely bioluminescent or confirmed*, genus-level record of bioluminescence or confirmed at species-level; NA: *unknown*, no record of bioluminescence at species or genus level; electronic supplementary material, table S1). Depth ranges were assigned to each taxon as follows: deep (greater than 200 m) or shallow (less than 200 m)—documented by over 90% of occurrences for the genus being found in a depth zone, or shallow/deep (spanning both deep and shallow ranges), according to OBIS (https://obis.org; electronic supplementary material, table S1).

### (b) Bioluminescence across Octocorallia

#### (i) Phylogenomic analyses

A new fully resolved phylogeny for Octocorallia reconstructed using target-capture sequence data (from 739 loci from 185 octocoral taxa) [19] was used to explore the evolution of bioluminescence in octocorals. The tree was constructed using maximum likelihood in IQTree v.2.1 [20] using the best model (GTR + F + R10) determined by ModelFinder [21]. Ultrafast bootstrapping (-bb 1000, [22]) and the Sh-like approximate likelihood ratio test (-alrt 1000, [23]) were conducted. For further details on sequence data acquisition and tree building methods, see McFadden *et al*. [19].

Divergence-dating of this recently revised octocoral tree was conducted using BEAST v. 2.6 [24] on the Smithsonian High-Performance Computing Cluster Hydra (doi:10.25572/SIHPC), as outlined in Quattrini *et al*. [10]. First, we generated a time-calibrated starting tree of the phylogeny in McFadden *et al*. [19] using penalized likelihood method (*chronopl*, based on [25]) in the R package *ape* [26]. The calibration points included the minimum ages of two fossils outlined in table 1 and a minimum age at the crown Octocorallia and at the root of the tree, obtained from [10]. We then selected a 25-locus alignment to input into the BEAST v.2.6 analysis [24]. The 25 loci (352–1419 bp alignments) were selected based on (1) a low risk of substitution saturation, as determined by entropy tests with *PhyloMad* [30,31], and (2) containing the highest number of individuals (greater than 85%) among all unsaturated loci. In the BEAST analysis, loci were partitioned so that a GTRGAMMA model (initial 1.0, 0 to infinity bounds) was applied to each locus. Exponential priors were used for fossil calibration points and a normal prior was applied to the crown Octocorallia and root of the tree, based on [10] (table 1). A relaxed clock model, with a lognormal distribution on the ucld.mean (initial 0.0002, 0-infinity bounds) and uniform distribution on the ucld.stdev (initial 0.1, 0–1 bounds), were applied. A birth–death tree prior was also used, with uniform priors on the birth rate (initial 1.0, 0–1000 bounds) and death rate (initial 0.5, 0–1 bounds). Two separate runs of 200 M generations were conducted. Log and tree files from each run were combined in LogCombiner [32], with a 10% burnin. The combined log file was assessed for convergence of parameter values and age estimates by inspecting traces and effective sample sizes in Tracer v.1.7 [33]. Trees were combined from both runs, and resampled by selecting one out of every 10 K trees, resulting in ~75 K trees. TreeAnnotator [32] was then used to produce a maximum clade credibility tree based on mean ages. An analysis (200 M generations) was also conducted without data by Sampling from the Prior, in order to ensure that the results were driven by the data and not solely by the prior information. This phylogeny includes robust age estimates and highly supported phylogenetic relationships across phenotypically diverse clades which enabled the examination of bioluminescent character evolution across deep time. The resulting phylogeny also differed slightly from the phylogeny in McFadden *et al*. [19], likely due to loci used in dating analyses; however, the topology was also recovered previously in McFadden *et al*. [11].

**Table 1.** Prior information on calibration points used in divergence dating analyses.

fossil	mean + offset (Ma)	mean + s.d. (Ma)	age range (95%CI) (Ma)	prior type	reference
Helioporidae	1.36 + 136	—	136–186	exponential	[27,28]
Keratoisididae	3.4 + 16.7	—	16.8–29.2	exponential	[27,29]
Octocorallia	—	578 + 50	480–676	normal	[10]
root	—	770 + 62	648–892	normal	[10]

We expanded the highly supported tree generated using ultraconserved elements (UCE tree) in [19], by combining mitochondrial data, mutS-like DNA repair gene (mtMutS), for an additional 107 octocoral taxa [19] to produce a hybrid (UCE/MutS) phylogeny. This hybrid tree contains a more comprehensive taxonomic coverage than the UCE tree alone, namely for known bioluminescent genera. Phylogenomic analyses were conducted using maximum likelihood and a partitioned analysis for multi-gene alignments with IQTree v.2.1 using both the concatenated UCE alignment (739 loci) and the alignment for mtMutS [20]. ModelFinder was used to find the best substitution model for each partition (UCE/mtMutS; electronic supplementary material, table S2). The partition model [29] was given discrete substitution models for each gene/character set and each partition was allowed to have its own evolution rate. Ultrafast bootstrapping (-bb 2000) and the Sh-like approximate likelihood ratio test (-alrt 1000) were conducted. The reconstructed tree was pruned using the phytools (v.1.2-4) [34] package in R (v.3.5.0) [35] to remove four aberrantly placed taxa with poorly supported nodes, based on the recently published phylogeny [19]. Polytomies were subsequently removed based on the following criteria that was: branches included (1) non-bioluminescent taxa with low bootstrap support, (2) a single taxon only included in the mtMutS dataset, or (3) a clade of taxa adjacent to known bioluminescent taxa. This resulted in the removal of 17 additional octocoral taxa from the final hybrid (UCE/MutS) octocoral tree (=270 operational taxonomic units or OTUs). The Scleralcyonacea clade was rooted to the Malacalcyonacea clade for downstream analysis using the *pxrr* program in the phylogenetic tool package Phyx [36] based on the findings of McFadden *et al*. [19].

### (c) Ancestral state reconstruction

Trait tables were generated for octocoral taxa represented in both the time-calibrated UCE tree (185 OTUs) and the UCE/MutS hybrid tree (270 OTUs) based on a scale of 0–1, as previously described (0: *non-bioluminescent or unlikely*; 1: *likely bioluminescent or confirmed*; NA or 0.5: *unknown*; see electronic supplementary material, tables S3, S4). Ancestral states of bioluminescence were calculated using stochastic character mapping, sampling ancestral states from posterior probability distributions [37] generated from 100 stochastic character maps for the bioluminescence trait using the *make.simmap* function (nsim = 100) in the R package phytools. An ‘equal rates’ model (= ‘ER’), assuming equal probabilities of trait gain and loss, the ‘symmetric’ model (= ‘SYM’), where the rate of change between the two states is the same forward as it is backwards, while accounting for unequal frequencies, and an ‘all rates different’ model (=‘ARD’), were used to estimate the evolution of bioluminescence in Octocorallia. Because ancestral state reconstruction (ASR) methods can potentially produce over- or under-estimates depending on assumptions taken regarding the probability of trait gain/loss (as reviewed in [5]), the best-fit model was determined by fitting and comparing the extended Mk models (*fitMk*) for discrete character evolution [38]. Akaike information criterion (AIC) values for the fitted models were then compared to determine the model with the lowest AIC score. This was found to be the ARD model assuming different rates of trait gain/loss, which was used for downstream analysis. Phytools was then used to plot one stochastic character map for the bioluminescence trait on both the time-calibrated phylogeny for Octocorallia (UCE tree) and the more inclusive hybrid tree, along with the posterior probabilities (pie charts) at each node. *corHMM*: hidden Markov models of character evolution [39], which allows for different transition rates across the phylogeny, was further used in R to estimate ancestral states using the rayDISC package, which treats NAs as missing data (according to [40]). These estimates supported those found with *fitMk*, which are the primary focus of downstream analysis.

### (d) Ancestral depth range estimation

To estimate the ancestral depth ranges, a Bayesian dispersal–extirpation–cladogenesis (DEC) model [41] implemented in RevBayes [42] was conducted on the dated, maximum clade credibility tree. Ancestral ranges of shallow (less than 200 m) and deep-sea (greater than 200 m) depths were estimated following the guidelines in the online tutorial on simple analysis of historical biogeography (https://revbayes.github.io/tutorials/biogeo/biogeo_simple.html). Current depth ranges were coded as binary values (0,1) denoting whether a species was recorded from deep, shallow, or both depth ranges. Depths were obtained from obis.org and supplemented with recent museum (National Museum of Natural History) and geome (https://geome-db.org) records and the literature (e.g. [43,44]), as previously described. For species identified to a rank above genus, the depth of collection was used. In RevBayes, the number of generations (MCMC) was set to 5000. The R package *RevGadgets* [45] was then used to plot the probability of ancestral states (as pie charts) at the nodes.

### (e) Depth distribution of bioluminescent octocorals

Depth ranges for extant octocorals were derived from the historical video annotations of 621 remotely operated vehicle (ROV) dives by the Monterey Bay Aquarium Research Institute between 1991 and 2016. A subset of anthozoans out of the 139 000 benthic annotations was cross-referenced to a database of bioluminescence capabilities, as described in [15], and the depth distributions were visualized using R.

## 3. Results

**Figure 1.**
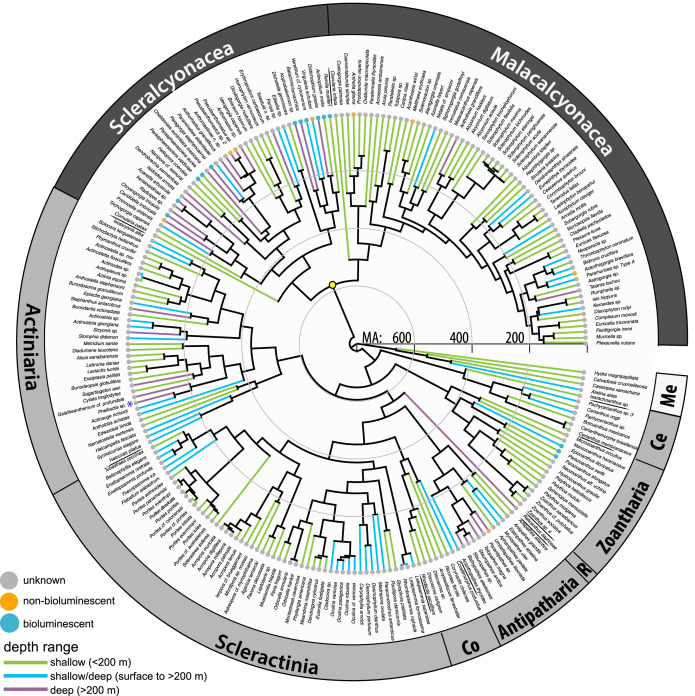
Phylogeny of Anthozoa with bioluminescent states and depth ranges. Time-calibrated tree adapted from Quattrini *et al*. [10] based on UCE data, with names updated based on taxonomic revisions to the World Register of Marine Species (WoRMS) or updated octocoral classifications from McFadden *et al*. [19]. Classes are colour coded (dark grey bars, Octocorallia; light grey bars, Hexacorallia) and orders are indicated (Ce, Ceriantharia; Co, Corallimorpharia; R, Relicanthus). Medusozoan (Me) outgroup is indicated in white. Taxon branches are colour-coded based on known genus-level depth ranges (green, shallow < 200 m; purple, deep > 200 m; blue, shallow/deep range spans both). Yellow circle indicates the estimated divergence point for Octocorallia. Asterisk indicates that bioluminescence has been documented in a closely related species.

Among Anthozoa, bioluminescence is found in both major classes that diverged approximately 770 million years ago (Ma). For Hexacorallia, according to current observations, bioluminescence was only retained in the early-diverging orders Zoantharia and Actiniaria (figure 1, crown ages 430–515 Ma). But overall, few bioluminescence records have been noted in both of these sea anemone orders. To date, bioluminescence has not been recorded from the orders Ceriantharia, Antipatharia, Corallimorpharia and Scleractinia, although one dubious record exists from an unidentified Antipatharia [1] and a luciferase-like gene was found in a ceriantharian [12].

**Figure 2.**
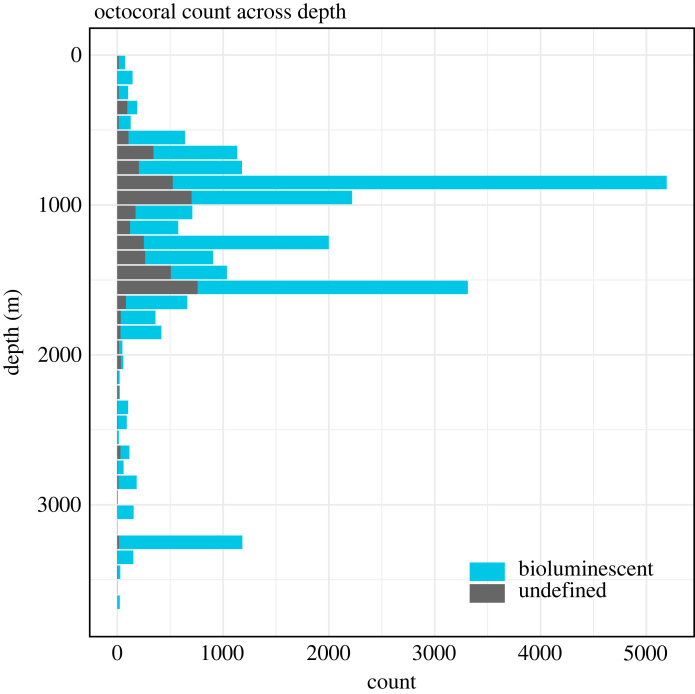
Octocoral abundance across depth. Depth distributions of octocorals observed during ROV dives offshore of Monterey Bay, California. Species were categorized based on the trait table as likely to be bioluminescent (species- or genus-level observation) or undefined (no record), and those distributions are plotted as stacked bars.

Bioluminescence is most prevalent among Octocorallia, particularly in species within the order Scleralcyonacea that resides in deep waters (figure 1). Despite dubious records of bioluminescence in Malacalcyonacea (i.e. *Alcyonium* sp. (Mangolds 1910), unidentified *Eleutherobia* that are likely to belong to genus *Parasphaerasclera* (Williams 2001)), no confirmed records of this trait exist for extant species belonging to this order. Within Octocorallia, bioluminescence is prevalent across both shallow and deep habitats and appears most abundant between depths of approximately 500–2000 m (figure 2; electronic supplementary material, figure S2). An additional deep (abundance) maximum exists around approximately 3300 m.

Testing bioluminescence in anthozoans using live specimens reported here (table 2; electronic supplementary material, figure S1) provides new records for bioluminescence for two species from Hexacorallia and five species from Octocorallia, with five of them (*Bullagummizoanthus, Corallizoanthus, Echinoptilum, Metallogorgia* and Keratoisidinae sp.) being the first reports at the genus level, and a total of 14 negative records for non-bioluminescent species. We carefully investigated previous reports and categorized some reported bioluminescent taxa as unknown or non-bioluminescent taxa (footnote of electronic supplementary material, table S1). The updated list of luminous anthozoans (electronic supplementary material, table S5) contains eight genera from Hexacorallia and 32 genera from Octocorallia.

**Table 2.** Results for bioluminescence tests performed in this study. Positive results for previously published records of bioluminescent species are not listed. Note, negative results for bioluminescence might be affected by several factors such as sampling stress, seasonal and/or temporal changes in internal concentrations of luminescent materials.

family	species	bioluminescence
HEXACORALLIA		
Parazoanthidae	*Bullagummizoanthus emilyacadiaarum*	*+*
Parazoanthidae	*Corallizoanthus* sp*., C. tsukaharai*	*+*
OCTOCORALLIA		
Alcyoniidae	*Anthothela grandiflora*	−
Chrysogorgiidae	*Chrysogorgia tricaulis*	*+*
Chrysogorgiidae	*Chrysogorgia* cf. *chryseis*	*+*
Chrysogorgiidae	*Iridogorgia* cf. *splendens*	*+*
Chrysogorgiidae	*Metallogorgia* cf. *melanotrichos*	*+*
Coralliidae	*Paragorgia johnsoni*	−
Coralliidae	*Pseudoanthomastus* sp.	−
Echinoptilidae	*Echinoptilum macintoshi*	*+*
Euplexauridae	*Euplexaura crassa*	−
Keratoisididae	*Keratoisididae* spp. (Clades B1, D1, D2, I, J3, S1)	*+*
Melithaeidae	*Melithaea japonica*	−
Paramuriceidae	*Acanthogorgia aspera*	−
Paramuriceidae	*Acanthogorgia breviflora*	−
Paramuriceidae	*Acanthogorgia spissa*	−
Paramuriceidae	*Echinogorgia* sp*.*	−
Paramuriceidae	*Paramuricea* sp. Type A	−
Paramuriceidae	*Villogorgia* sp.	−
Plexauridae	*Swiftia exserta*	−
Primnoidae	*Candidella imbricata*	−
Virgulariidae	*Virgularia* spp.	−

### (a) Evolution of bioluminescence in Octocorallia

**Figure 3.**
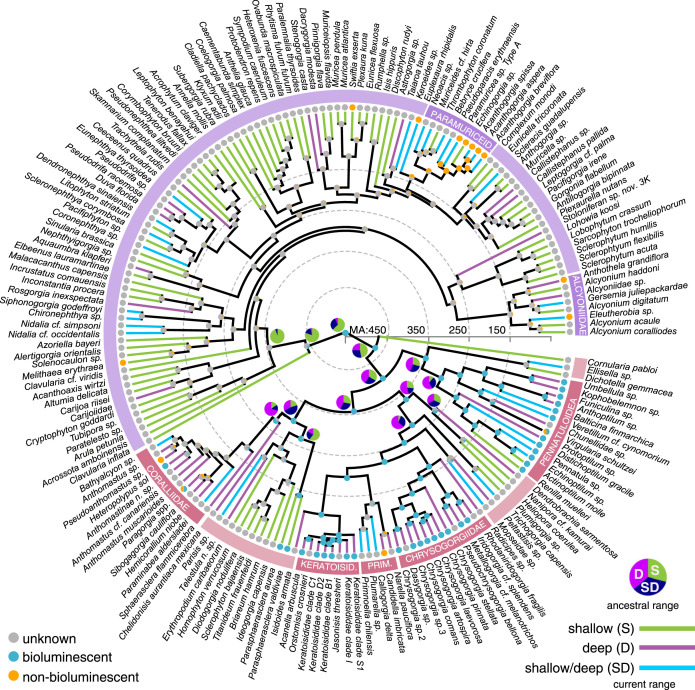
Phylogeny of Octocorallia with bioluminescent ancestral states. Newly time-calibrated tree adapted from McFadden *et al*. [19] using UCE data. Traits were defined as bioluminescent (species or genus-level record), non-bioluminescent (negative record) or unknown (no record). Taxon branches are colour coded based on known genus-level depth ranges (green, shallow < 200 m; purple, deep > 200 m; blue, shallow/deep range spans both). Ancestral depth range estimates are from electronic supplementary material, figure S4. The two major clades represent the orders Malacalcyonacea (purple) and Scleralcyonacea (pink).

Ancestral state reconstruction revealed that the most recent common ancestor of octocorals was bioluminescent (figure 3; electronic supplementary material, figure S3). This analysis places the origin of bioluminescence in Octocorallia to at least or just prior to the Cambrian era (542 Ma, 95% CI: 463–624 Ma), providing insight into how early this trait arose in Metazoa and its long-standing prevalence within octocorals specifically. Subsequently, putative losses of bioluminescence occurred across the class. The ancestral state of the order Malacalcyonacea, which diverged approximately 440 Ma (CI: 345–532 Ma) and comprises primarily soft corals, without calcitic skeletal axes, is currently unknown. Many octocorals in this order occur in shallow water, with the ancestral state and bioluminescent capabilities of an overwhelming majority of malacalcyonaceans being unknown (figure 3). However, unless bioluminescence was lost early in Malacalcyonacea, multiple losses may have occurred in this group, including in the most recent common ancestor (MRCA) of Paramuriceidae (figure 3).

The ancestral state for the order Scleralcyonacea (460 Ma CI: 376–542 Ma) was bioluminescent, with at least three confirmed losses in at least three families (Coralliidae, Primnoidae and Virgulariidae). The ancestral character states of the superfamily Pennatuloidea, and families Chrysogorgiidae and Keratoisididae were bioluminescent. These octocoral families had the highest number of bioluminescent representatives in this study: Pennatuloidea (*n* = 12), Chrysogorgiidae (*n* = 10) and Keratoisididae (*n* = 7) (electronic supplementary material, tables S3–S5). Results from broader taxonomic sampling (UCE/mutS tree, electronic supplementary material, figure S3) support the results from the limited (UCE) taxonomic sampling.

### (b) Ancestral depth range estimation

Depth range estimations in Octocorallia revealed that the MRCA of octocorals most likely resided in shallow waters (figure 3; electronic supplementary material, figure S4). The MRCA of the likely non-bioluminescent/unknown Malacalcyonacea clade was also a shallow-water ancestor. Interestingly, the MRCA of the largely bioluminescent Scleralcyonacea clade may have inhabited a broad depth range across both shallow and deep waters. With the sister lineage *Cornularia pabloi* excluded, the MRCA of the remaining Scleralcyonacea clade was most likely found in deep water or at intermediate depths (shallow/deep) (figure 3; electronic supplementary material, figure S4). The MRCA of the families (Keratoisididae, Chrysogorgiidae) and superfamilies (Pennatuloidea) that are largely bioluminescent had a high probability of inhabiting the deep sea (figure 3).

## 4. Discussion

This research provides insight into the timing of the emergence of bioluminescence in Metazoa and context for the evolution of this trait across anthozoan cnidarians. Our study shows that bioluminescence is present across Anthozoa and arose in Octocorallia at least by the Cambrian period. Our divergence dating placed the origin of Octocorallia approximately 542 Ma (figure 3), whereas Quattrini *et al*. [10] estimated the origin of Octocorallia in the Ediacaran (575 Ma, 95% CI: 484–685 Ma, figure 1). Nevertheless, our results provide evidence that octocorals, among currently known luminescent species, represent the oldest lineage with species that exhibit bioluminescence. Previously, the oldest documented origin of bioluminescence was *ca* 267 Ma in the Permian period in a group of crustaceans [7]. It is possible, however, that bioluminescence had even earlier origins, as it is present not only within hexacorals, but also within many ctenophores, the sister lineage to all other metazoans [46]. Further investigations into the luciferase(s) driving bioluminescence is vital to understanding the emergence and homology of this trait. If bioluminescence served an adaptive role in deep time, organisms with eyes or photoreceptors would also have been necessary, regardless of whether the luminescence was for symbiosis or defense. Since creatures with eyes and other light-sensitive photoreceptors had already appeared in the Cambrian era [47], it is plausible to think that communication using light between anthozoans and other photoreceptor-bearing organisms was in place at that time. Regardless of the role of this trait throughout time, our study provides evidence that bioluminescence has much earlier origins than previously recorded.

Given the reliance of bioluminescent reactions on oxygen, it has been previously speculated that bioluminescence initially evolved to eliminate oxygen or reactive oxygen species (ROS) from an organism as a detoxification strategy, with light production as a secondary by-product [48–50]. Selinger [51] hypothesized that bioluminescence was co-opted from an oxygen detoxification mechanism to a light-based form of communication. The levels of oxygen in the shallow oceans, although variable, began to increase prior to and during the Cambrian period, where the deep oceans experienced pervasive to periodic anoxia throughout geological time [52,53]. Given the paleoclimate conditions, it is plausible that bioluminescence arose early on in Octocorallia (and Anthozoa) as a defense mechanism against free-radicals but later became functionally useful as a form of communication with other light-sensing biota. Further, *Renilla*-type luciferases found among octocorals are thought to be homologous to bacterial haloalkane dehalogenases (hydrolase enzymes) [54,55] and possibly acquired through horizontal gene transfer events [55,56]. Haloalkane-dehalogenase function is hypothesized as the metazoan ancestral state [55] and co-opted to luciferases in specific cnidarian lineages, though homologues have not been recovered in hexacorals except the early branched lineage of Ceriantharia. Bioluminescence might have evolved in Anthozoa via co-optation of other homeostatic functions (e.g. antioxidant) and became more widespread as species began colonizing the deep sea, where light irradiance and metabolic activity are reduced (= lower endogenous ROS; discussed in [48]).

In our ASR analysis, many species were categorized as ‘Unknown’ for bioluminescence. This is due to the scarcity of information on species with bioluminescent traits relative to the diversity of Anthozoa; even less information is available about species that do not exhibit bioluminescence, which we term as non-bioluminescent, because negative observations like those described here are rarely reported. Consequently, our analysis is predicated on non-exhaustive trait states, marking a limitation in the current study. In this study, bioluminescence was detected via dark-adapted naked eye and digital cameras (e.g. Sony alpha 7sII). It is possible that luminescence is more readily detectable using other methodologies (e.g. luminometer) and that detection may vary by species. Therefore, it is important to note that false negatives for bioluminescence are possible, and even more so given the physiological stress associated with collections. Regardless, the negative records reported here represent less than 8% of the octocoral taxa in the ASR analyses. Changing these negative records to positive would only reinforce the bioluminescent ancestral state of Octocorallia reported here. To further assess whether the ancestral state was potentially overestimated towards bioluminescence due to the limited reports on non-bioluminescent species, we conducted analyses treating the ‘unknown’ category as non-bioluminescent. Still, similar results were obtained, reinforcing the hypothesis that the MRCA of octocorals was likely bioluminescent (electronic supplementary material, figure S5).

Alternative phylogenetic topologies could also have an impact on ASR inferences. The phylogeny presented herein is the most robust to date, with the majority of families represented, but it includes only a portion of all known octocoral taxa; and it is well known that inadequate taxon sampling and locus selection can impact phylogenetic inference (see [57]). The resulting topology also differed slightly from the phylogeny in McFadden *et al*. [19], with the Carijoidae and Acanthoaxiidae clades placed near the most early-diverging lineages, a topology more similar to McFadden *et al*. [11]. Nevertheless, our main findings remain the same when running ASR on the hybrid UCE/MutS tree (electronic supplementary material, figure S2), a topology incongruent with the phylogenomic one (UCE only) and containing 107 additional species. Taxon sampling might also impact the character states linked to the depth. Therefore, depth categories were generated based on genus, not species, to limit bias in the ancestral depth analyses.

Considering the difficulty of accessing deep-sea ecosystems and observing bioluminescence, the results summarized here are likely an underestimate of bioluminescent capabilities among Anthozoa. Rather, this study provides a conservative view on the evolution of bioluminescence among these basal metazoans. Current knowledge gaps in anthozoan bioluminescence summarized here provide a map to guide further documentation of this trait, or lack thereof, in both shallow- and deep-water environments. Our findings also indicate that taxa with unverified bioluminescent traits belonging to families with bioluminescent ancestors (e.g. Pennatuloidea, Chrysogorgiidae, Keratoisididae and Coralliidae) are promising targets for future investigations into the bioluminescent capabilities of Anthozoa.

Previous studies [12] have identified luciferase-coding genes in bioluminescent octocorals and their homologues from non-bioluminescent octocorals. Luciferase is the enzyme directly involved in producing light in an organism and sequence comparisons have proven exceptionally useful to understanding bioluminescent capabilities in a variety of systems [58,59]. The presence of luciferase among a diverse array of octocorals across both orders [12] supports our findings that the common ancestor of Octocorallia was bioluminescent, with the trait lost as a result of other contributing factors (e.g. drift or selection) during the subsequent evolution of various lineages. However, the functional verification of these luciferases is limited. Where direct observation of bioluminescence is not feasible, genera and/or species predicted to be bioluminescent in this study can be opportunistically confirmed via immunoreactivity against the *Renilla*-type luciferase as samples become available, as has been done in prior studies on a handful of sea pens (e.g. [12]).

The bioluminescent reaction also requires the chemical substrate coelenterazine. While many marine luminous organisms, including jellyfish, rely on coelenterazine and coelenterazine-dependent luciferases system, only a few species can produce coelenterazine independently: e.g. ctenophores and copepods [60,61]. Others, such as hydrozoan jellyfish and brittle stars, acquire coelenterazine through their diet [17,18]. The ability to synthesize coelenterazine in luminous octocorals and other cnidarians remains unknown. An alternative hypothesis is that octocorals cannot synthesize coelenterazine but depend on a dietary source. But if the MRCA of Octocorallia was the first coelenterazine-dependent bioluminescent animal in the ocean, then the question arises: what was the provider of the coelenterazine? Further research is needed to test these hypotheses and to understand these ancient bioluminescent organisms and the origin of bioluminescence from both evolutionary and ecological perspectives.

While the emergence of bioluminescence in the MRCA of Octocorallia does not appear to be associated with deep-water ancestry, our results suggest the MRCA of the largely bioluminescent Scleralcyonacea had an expanded depth range across shallow to deep waters, with the trait largely maintained in descendants of deep-water ancestors. This suggests that retention of bioluminescence within this group is associated with a deep-sea lifestyle or perhaps that the presence of this trait is what enabled species to diversify throughout the deep sea. All of the octocoral families that have multiple records of bioluminescent species evolved from bioluminescent, deep-sea ancestors. Our data also suggest that many of the bioluminescent genera that occur in shallow water evolved from bioluminescent MRCAs likely inhabiting the deep sea, indicating the emergence of bioluminescent, deep-water lineages into shallow-water habitats. Further investigation is needed to elucidate both the genomic and environmental factors contributing to bioluminescent trait loss/conservation over time in Anthozoa.

Bioluminescence is not as prevalent in Hexacorallia, which may have evolved a different genetic basis for light emission. Among hexacorals, bioluminescence is primarily maintained in the early-diverging order Zoantharia, which lacks skeletons—the predicted ancestral skeletal state of Anthozoa [10]. Interestingly, bioluminescent zoanthids are often found living on coral and sponge species that house invertebrate associates, while non-bioluminescent zoanthid species are more commonly found growing on carbonate substrates (A.M.Q. 2016, pers. observ.). Communication with commensals and defense against predators have been suggested as putative roles for bioluminescence among anthozoan cnidarians (e.g. [4,16]), although the true nature of these ecological associations remains enigmatic. Further investigation into the adaptive value of this trait in Anthozoa may shed light on the scarcity of this trait in hexacorals.

## Data Availability

Code to accompany the analyses conducted in this study can be found on Dryad (https://doi.org/10.5061/dryad.37pvmcvsj), along with other supporting data files including tables, alignments, log and tree files [62]. Supplementary material is available online [63].
